# Isolated third cranial nerve palsy as the presenting sign of tuberculum sellae meningioma: a case report

**DOI:** 10.3389/fopht.2025.1525183

**Published:** 2025-04-30

**Authors:** Azizah Alotaibi, Fay Althunayyan, Shuroq Alshehri, Hosam Al-Jehani

**Affiliations:** ^1^ Department of Neurosurgery, King Fahd University Hospital, Imam Abdulrahman Bin Faisal University, Al Khobar, Saudi Arabia; ^2^ College of Medicine, King Fahd University Hospital, Imam Abdulrahman Bin Faisal University, Al Khobar, Saudi Arabia; ^3^ Department of Neurology and Neurosurgery, Montreal Neurological Institute, McGill University Health Centre, Montreal, QC, Canada; ^4^ Department of Neurosurgery, Weill Cornell University, Houston Methodist, Houston, TX, United States

**Keywords:** meningioma, corticosteroids, oculomotor nerve, tuberculum sellae meningioma, neurophthalmology

## Abstract

Tuberculum sellae meningioma (TSM) is an uncommon tumor among all intracranial meningiomas. As these tumors grow, they compress the surrounding structures, including the optic nerves and the pituitary gland. Ocular motor nerve palsy (OMNP) can occur as an isolated mononeuropathy or as part of multiple cranial nerve palsies. The role of corticosteroids in the management of OMNP has not been fully studied in the literature. In this report, we present a case of a previously well middle-aged woman who presented with severe headache and isolated OMNP on examination. MRI of the brain showed a small TSM that extends into the right optic canal. In our case, we noted the expedient and complete recovery of isolated OMNP within a few days following treatment with dexamethasone. This case report is on an isolated OMNP associated with TSM, which has not been previously reported. In addition, it highlights the role of corticosteroids in achieving rapid recovery from OMNP.

## Introduction

Tuberculum sellae meningioma (TSM) accounts for 5%–10% of all meningiomas. These tumors present a challenge due to their proximity to the optic nerve and the internal carotid artery, as well as the hypothalamus, infundibulum, and the pituitary gland. TSM typically presents with visual impairment, most commonly a decrease in visual acuity and defects in the visual field. Acquired oculomotor nerve palsy (OMNP) is a condition that could occur in isolation or as part of multiple cranial nerve palsies. Patients usually present with headaches, retro-orbital pain, and the classic “down and out” positioning. The various etiologies of isolated OMNP include microvascular ischemia, traumatic injury, and compression by a neoplasm or aneurysm, among others. Herein, we present a case of a female patient who presented with an isolated OMNP in the background of TSM.

## Background

A 57-year-old woman with a previous history of right frontal meningioma, resected years previously, presented to the emergency department with a chief complaint of a headache that started 6 days previously and was described as “the worst headache of my life.” It was associated with right-eye ptosis and retro-orbital pain. The patient denied any history of seizures, nausea, or vomiting. She also denied a history of conjunctival congestion. Her past medical history was unremarkable apart from a motor vehicle accident, which was around 20 years ago. CT of the brain at that time was unremarkable.

**Figure 1:**
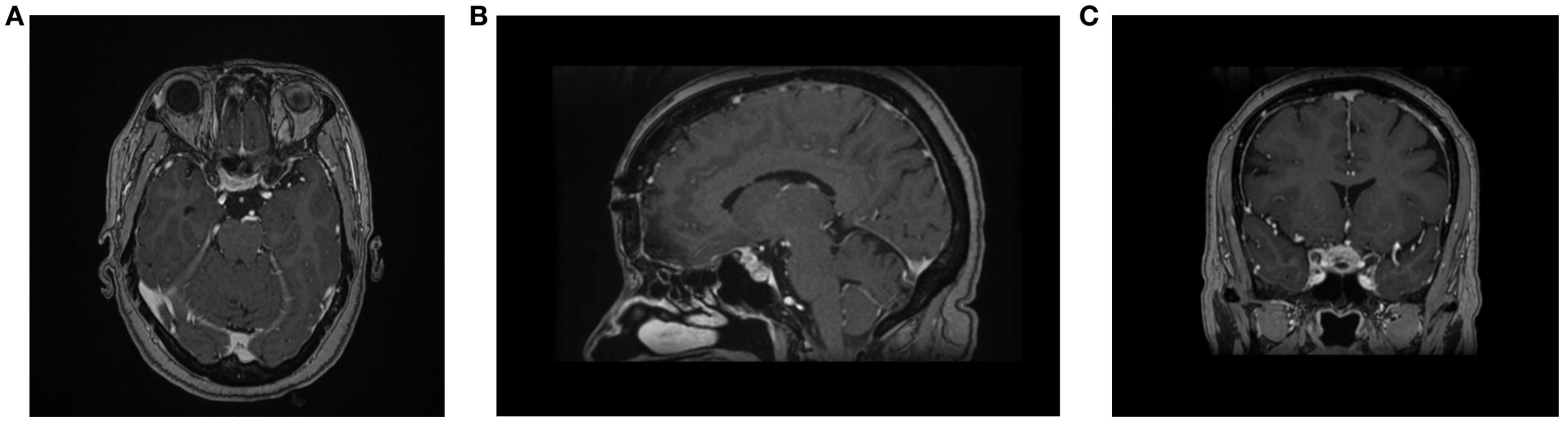
**(A)** Axial, **(B)** coronal, and **(C)** sagittal contrasted MRI showing a small (0.8x0.8 cm) tuberculum sellae meningioma that extends into the right optic canal.

**Figure 2:**
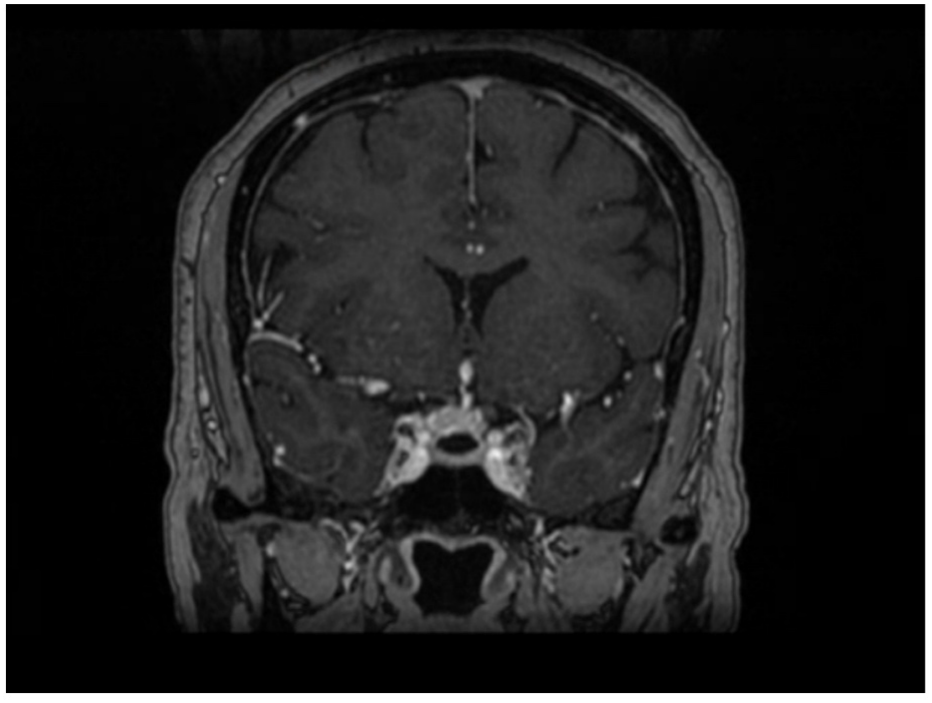
Coronal contrasted MRI showing no involvement of the cavernous sinus.

Upon neurological examination, the patient was conscious, alert, and oriented to time, place, and person. The examination was remarkable for a 4-mm sluggish, reactive right pupil and ptosis of the right eye, as well as limitation of adduction and an upward gaze with preserved abduction. Examination of the left eye was unremarkable. CT of the brain was performed, which showed a small partially calcified TSM. CT angiography showed no evidence of aneurysm or significant stenosis. MRI of the brain showed a small avid enhancing extra-axial lesion seen within the tuberculum sellae more into the right side, which appeared isointense on T1- and T2-weighted imaging with extension anteriorly as a dural tail on the planum sphenoidale as shown in **Figures 1**, **2**. The lesion extends to the optic canal and abuts the intracanalicular segment of the right optic nerve. There was no evidence of extension to the pituitary gland or sphenoid sinus. The lesion measured 0.8 × 0.8 cm. There was no evidence of other brain lesions. Multidisciplinary discussion was conducted. Surgery was deferred given the small size of the lesion, and radiosurgery was thought to be of delayed benefit for such an acute presentation of acute nerve palsy.

The patient was started on intravenous dexamethasone 6 mg and continued dexamethasone 3 mg every 8 h. Interestingly, within 4 days of follow-up, she had an immediate resolution of her symptoms, with no evidence of third cranial nerve palsy on examination. The patient continued dexamethasone for 5 more days and was discharged home with a marked improvement on dexamethasone tapering, with an outpatient follow-up in ophthalmology and neurosurgery clinics.

## Patient perspective

The symptoms I had were very bothersome for me. When I sought medical help at King Fahad University Hospital, I was grateful to the treating team for explaining my condition thoroughly and discussing treatments. At first, I was disturbed by the severity of my headaches and visual changes, but the treating team provided me with reassurance, and my symptoms have thankfully improved after the medication. I understand and consent that information regarding my condition might be used for research purposes.

## Discussion

TSM commonly presents with visual symptoms such as a decreased visual acuity and hemianopsia. Based on their location, these tumors are classified as suprasellar lesions, which also include diaphragm sellae meningiomas (1). These tumors typically cause optic nerve compression and become symptomatic early in the course of the disease, requiring prompt treatment directed at preserving and improving vision. In a study that examined the epidemiology and clinical profile of TSM, which included 36 patients, approximately 89% had a reduction in visual acuity, whereas hemianopsia was found in 50% of patients (2). It also found that canal invasion was associated with visual impairment symptoms and that both canal and vascular invasion can impact the respectability of the tumor (2). Magill et al. reported preoperative visual deficits present in 87% of patients, in addition to describing that a higher tumor score was associated with a higher risk of visual worsening (3). In a study by Lee et al., which examined the anatomical origin of TSM, 89% of patients had marked asymmetrical visual field defects (VFDs), while 6% had symmetric VFDs (4). In addition, optic canal involvement was observed in 73% of patients (4). The authors reported that the preoperative symptom duration, the laterality of the origin of TSM, and optic disc atrophy were associated with long-term poor visual outcomes (4).

OMNP is a neuro-ophthalmological condition that can occur in isolation as a mononeuropathy or as a multi-neuropathy where multiple concomitant cranial nerve palsies are involved. Several studies have examined the underlying etiologies of acquired OMNP, which include microvascular ischemia (42%), followed by trauma (12%), compression by neoplasm (11%), iatrogenic or post-surgical (10%), and compression by an aneurysm (6%) (5).

TSM can affect cranial nerves in the vicinity, including the third cranial nerve (6). In our search of the English literature, we were not able to identify any previous publication of isolated OMNP with TSM. In our case, the patient presented with an isolated third cranial nerve palsy, with no other visual findings. Interestingly, the use of corticosteroids in this case was shown to expedite recovery and to restore significant and complete function in a short time.

The role of corticosteroids in the management of OMNP has not been fully studied. In the very few reports in the literature, improvement of symptoms following corticosteroid use took approximately 2 months. In the present case, we noted the expedient and complete recovery from isolated OMNP within a few days following the administration of corticosteroids. Morisaki et al. described the effect of corticosteroids on Schwann cells by increasing the expression of glucocorticoid receptors (GRs), improving myelin formation *in vitro*, which possibly explains the clinical improvement (7).

In one study, a 10-year-old boy with coronavirus disease 2019 (COVID-19) infection who was treated with prednisolone (2 mg/kg per day for 10 days) showed a marked improvement in ptosis and diplopia by day 3 and complete recovery by day 7 of treatment (8). Another case involved a 40-year-old man who was previously healthy and who presented with left partial OMNP, which was assumed to be secondary to viral microvascular injury. He received oral dexamethasone 8 mg every 8 h for a total of 7 days. It was noted that, within a week, the left eye mobility began to return; in a total of 3 months, the patient had complete resolution of symptoms and had a normal ophthalmologic examination (9).

Isolated OMNP has been described in a single report in the literature in association with a parasagittal meningioma (10) and in another as the presenting sign of a posterior fossa meningioma (11). To our knowledge, isolated OMNP has not been previously reported in association with TSM.

## Concluding remarks

TSM constitutes one of the common intracranial tumors. Most patients typically present with visual acuity impairment and, to a lesser extent, VFDs. Preoperative visual deficit is one of the key factors that predict outcomes, emphasizing the role of early surgical treatment. Isolated OMNP has been previously reported in the literature, but not in the background of TSM. This report also highlighted the effectiveness of corticosteroids in achieving rapid and complete recovery from OMNP.
